# Clinical Validation of Computer-Assisted Navigation in Total Hip Arthroplasty

**DOI:** 10.4061/2011/171783

**Published:** 2010-11-04

**Authors:** Eric Beaumont, Pierre Beaumont, Daniel Odermat, Isabelle Fontaine, Herbert Jansen, François Prince

**Affiliations:** ^1^Laboratoire de Recherche en Orthopédie, Hôpital du Sacré-Cœur de Montréal, 5400 Boulevard Gouin Ouest, Locales K-3115, Montréal, QC, Canada H4J 1C5; ^2^ORTHOsoft Inc., Computer Assisted Surgery, 75 Queen Street, Suite 3300, Montréal, QC, Canada H3C 2N6; ^3^Département de kinésiologie, Université de Montréal, 2100 Édouard-Montpetit, Montréal, QC, Canada H3T 1J4

## Abstract

A CT-based navigation system is helpful to evaluate the reamer shaft and the impactor position/orientation during unilateral total hip arthroplasty (THA). The main objective of this study is to determine the accuracy of the Navitrack system by measuring the implant's true anteversion and inclination, based on pre- and postoperative CT scans (*n* = 9 patients). The secondary objective is to evaluate the clinical validity of measurements based on postop anteroposterior (AP) radiographs for determining the cup orientation. Postop CT-scan reconstructions and postop planar radiographs showed no significant differences in orientation compared to peroperative angles, suggesting a clinical validity of the system. Postoperative AP radiographs normally used in clinic are acceptable to determine the cup orientation, and small angular errors may originate from the patient position on the table.

## 1. Introduction

The orientation of the acetabular component has been shown to be a significant factor affecting the risk of dislocation, impingement, and wear between components in patients undergoing total hip arthroplasty (THA) [1–3]. The prevalence of implant dislocation following THA ranges between 1% and 5% and represents a significant cause of early failure [4–7]. In addition to dislocation, implant impingement causes excessive wear of the cup liner through the impaction of the neck and creation of debris, a contributor to implant loosening via bone resorption. Therefore, a proper implant positioning is essential in order to reduce the incidence of impingement and the risk of associated complications. Despite the availability of many techniques of stabilizing and positioning the pelvis during the surgical procedure, many surgeons admit that it is difficult to know precisely how the patient's pelvis is oriented during surgery. This may lead to improper cup placement when using mechanical guides [8–10]. To improve this particular phase of the surgery, a navigation system was designed to help surgeons to evaluate the reamer and the impactor position/orientation during surgery. This system's precision is crucial because it can have a direct effect on the final cup placement [11, 12]. Several validation studies using plastic bone models and cadavers were undertaken by Orthosoft Inc. to assess the system's accuracy, which demonstrated a global error in cup orientation (<2° in 95% of cases and <5° for 99% of cases, unpublished data). To evaluate the system's accuracy with patients, data was gathered to estimate the system's validity. In this regard, the precision and validity of the “Navitrack” system at inserting pedicle screws has already been established [13].

The first aim of this study was to compare the cup orientation angles provided by the Navitrack navigation system and the true cup position. A second objective was to compare the cup orientation measured on standard AP radiographs to the cup orientation measured from the postoperative CT-scans. This would allow estimating the discrepancy of the cup orientation from 2D radiographs, which is a standard postoperative validation method used in clinic.

## 2. Materials and Methods

The clinical validation was conducted on 9 patients undergoing unilateral THA. The average patient age was 58 years old (range 46–77 years); including 1 woman and 8 men. The affected hip was on the right side in 5 cases and on the left side in 4 cases. The Converge acetabular cup from Zimmer was positioned during surgery using the Navitrack Total Hip Replacement (THR) 1.3 system. This protocol was approved by the Sacré-Coeur hospital research ethics committee, all patients were informed about this protocol before their surgery, and they signed a consent form. 

**Figure 1:**
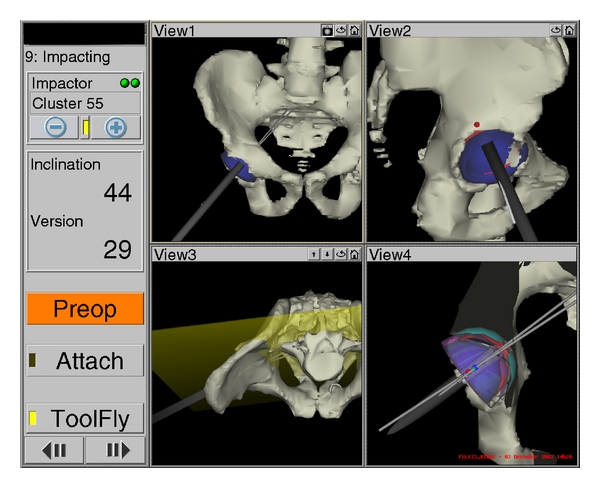
Representation of the preop cup placement (red) and the real cup position (blue) during the impacting process. An actual inclination of 44° and an anteversion of 29° are shown from an AP view (top left), a right side view (top right), and a top view including a yellow cutting plane that permit the visualization of the posterior portion of the pelvis (bottom left) and the planned cup (red) and navigated cup (blue) (bottom right).

The navigation system includes a software module to reconstruct the bone model of each patient based on computer tomography-scan (CT-scan) data. The preoperative CT-scan was matched with the intraoperative position of the patient's pelvis using a surface registration process. The patient's pelvis model was registered in space with a surface matching algorithm by using a pointer device equipped with reflecting spheres that can be tracked by an optical localization system (Polaris, Northern Digital Instruments). Reflective spheres were also used on a reference fixed on the iliac crest during the registration process, permitting the pelvis localization on the operating table. In addition, reflective spheres were attached to the acetabular reamer and the cup impactor, enabling their real-time tracking position during the procedure as shown in Figure 1. Furthermore, after the reaming process using the navigated reamer, the surgeon used the Zimmer standard mechanical impactor to position the cup. Next, the mechanical impactor was unscrewed without moving the acetabular cup and replaced by the navigated impactor to measure the orientation and to reposition the cup as needed.

For all surgeries performed in this study, the acetabular cup size and orientation were predetermined from the preoperative planning module using the Navitrack system. The preoperative cup size, position, and orientation within the 3D reconstructed pelvis are displayed during the surgery as shown in Figure 1. Postoperatively, a scan was performed, and a 3D model including the pelvis and the cup was built, in order to determine the real cup orientation. Secondly, postoperative AP radiographs were taken, and films were digitized using a vertical scanner to evaluate the final position of the cup. Technical details concerning the validation process of CT-scans and radiographs are described below.

### 2.1. Clinical Validation Using CT-Scans

**Figure 2:**
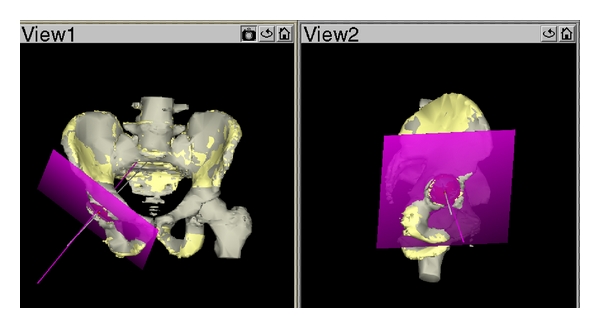
Example of a CT-scan validation process, where both models (pre- and postoperative) are superimposed to calculate the cup orientation based on the coordinate system defined preoperatively.

The CT-scan data was compared to the final navigation angles (system values) obtained during the surgery using the THA Navitrack system, which relates the cup position to the pelvic frontal plane (i.e., the plane defined by the bilateral anterior superior iliac spines and the anterior pubic tubercles). A postoperative scan was obtained from every patient within five days after surgery, to create a 3D image of the pelvis including the cup. In order to compare the pre- and postoperative models, an algorithm was developed, using several common landmarks on both 3D pelvis bone models, to virtually match the 3D models. The preoperative model includes the pelvis and the exact position of the coordinate systems that was established before surgery. Therefore, once both models are superimposed, the orientation of the cup (inclination and anteversion) is determined using a vector perpendicular to the plane defined by the peripheral ring (equator) of the cup using the same coordinate system than the intra-op 3D model. An example of the validation process is shown in Figure 2.

### 2.2. Validation Using Planar Radiographs

**Figure 3:**
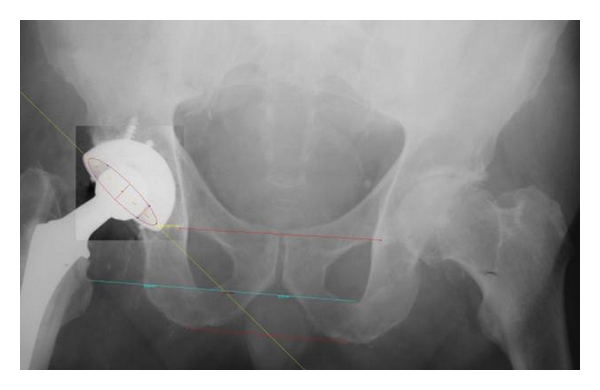
Digitalized representation of an AP radiograph centered over the pelvis as well as the determination of the inclination and anteversion using the software Imagika 1.50. To determine the anteversion, the ratio of the major and minor ellipses was used, which corresponds to the dark portion of the cup using the method of Ackland. The inclination of the cup was determined by measuring the angle between a line joining the ischial tuberosities or the teardrops and a line through the long axis of the ellipse.

Postoperative radiographs centered on the pelvis were taken, and the films were digitized using a vertical scanner (Vidar, Diagnostic Pro Plus). The Imagika 1.50 software was used to measure the cup orientation as shown in Figure 3. To determine the anteversion, the ratio of the major and minor ellipses was used, which corresponds to the dark portion of the cup according to the method of Ackland [14]. The inclination of the cup was determined by measuring the angle between a line joining the ischial tuberosities or the teardrops and a line through the long axis of the ellipse. In addition, nomograms, which are used in the literature [15] to convert radiograph angles into anatomical angles, were used to compare the anteversion and the inclination values of postoperative radiographs with those of the Navitrack navigation system. The anatomical inclination is defined by the angle between the impactor and the cranio-caudal patient axis. The anatomical anteversion represents the angle between the lateral axis and the projection of the impactor axis on the transverse plane [16].

### 2.3. Statistical Analysis

A nonparametric Wilcoxon signed rank test was used to compare the differences in the angular orientations of the acetabular implants between the final cup placement during the surgery and the postoperative scan evaluations. This test was also used to compare the final cup orientation between the postoperative scans and the AP radiographs centered over the pelvis. The significance level was set at *P* < .05.

## 3. Results

### 3.1. CT-Scan Validation

The orientation of the acetabular cup measured using a navigation device was determined from postoperative CT-scans and AP radiograph images. Data from 9 patients are shown in Table 1. The planned cup orientation that was established before the surgery was 47 ± 2° in inclination and 22°± 2° in anteversion. This target cup orientation was the ideal position during the surgery. The average final cup orientation at the end of surgery was 48 ± 6° in inclination and 25°± 3° in anteversion. To validate the final position of the cup, postoperative 3D reconstructions of the pelvis and the cup were performed, using CT-scan images, to measure the actual cup orientation. The average inclination of 45 ± 5° and the anteversion of 24 ± 3° of the cup were not significantly different than per-operative final reading angles (*P* = .5).

### 3.2. Radiograph Validation from Postoperative CT-Scan

Postoperative measurements of cup orientation from AP radiographs centered over the pelvis showed average inclination of 46°± 5° and anteversion of 23°± 3° values, after the conversion to anatomical definition using the conversion table of Murray [15], that were not significantly different from the 3D reconstructed postoperative CT-scans (*P* = .5). 

## 4. Discussion

### 4.1. Postoperative CT-Scans Validation

 In this section, we have compared the final per-operative cup orientation to postoperative CT-scans. These results are consistent with previous studies performed on cadaver specimens (unpublished data). Therefore, it can be considered that this system is accurate with patients within an acceptable margin. In regards to our results, small discrepancies can be explained by system errors during the surgery (registration and tracking accuracy) or methodological errors inherent to the pre/postoperative model matching technique. One inherent problem of the Navitrack and all CT-based navigation systems during the surgery is the possible mismatching between the virtual 3D model and the patient's pelvis during the initial registration part. Possible reasons for this are related to the approximation of 2D scan images for those patients during the segmentation, because a part of the femoral head is often directly in contact with the wall of the acetabulum, which is not the case in intact hips seen in cadavers. Secondly, the removal of osteophytes during the femoral head dislocation can lead to a mismatch during the registration process. To solve this problem, a software upgrade has been developed which rejects points that are outside of the pelvis surface to insure a good matching between the patient's pelvis and those from the virtual 3D model.

### 4.2. AP Radiographs Validation

In regards to the planar radiographs, the measurement's errors are most likely attributable to the patient's position on the table, since pelvic tilt (flexion/extension or lateral tilt) can modify the cup projection on the film [17, 18]. To evaluate this, a cup was positioned in a plastic bone models using the Navitrack. Next, using an angle ruler, it was shown that a pelvic tilt of 10° (flexion/extension or lateral tilt) leads to an error of more than 8° on the AP radiographs in regards to the cup inclination and anteversion (data not shown). Compared to postoperative CT-scans, the results obtained from AP radiographs are accurate, but they are subjected to a greater error in determining the implant positioning. Our results are well in line with Babisch and coll., 2008, showing that the pelvis orientation is not precisely known during the AP radiograph and the determination of the cup orientation is thus approximated [18].

### 4.3. CT-Based Navigation Device

The first objective involved in this study was to validate the angles provided by the navigation system to position the acetabular cup prosthesis. By using a CT-based navigation system, the goal was to track the acetabular reamer and the cup impactor relative to the patient's 3D reconstructed pelvis. During the procedure, after the reaming process assisted by the tracking system, the Centerpulse (Zimmer) mechanical guide (not navigated) was used to position the cup. Next, the surgeon unscrewed the mechanical impactor and replaced it by the navigated impactor without moving the cup, in order to evaluate the error in determining the cup orientation with the mechanical guide. In all cases, the inclination value was correct within 2° of error. However, in some cases, the anteversion was under- or overestimated by 9°, which is important to consider and justifies the utilisation of the navigation system presented in this study [16]. In addition, it must be considered that the reaming process was achieved using the navigated reamer, which facilitates the ideal cup orientation through the use of the mechanical impactor. Therefore, our results proposed that the implant positioning using a navigation system facilitates the surgeon's control of the cup anteversion and can help reducing variability in implant positioning. The lack of precision resulting from the use of mechanical guides may result in placement of the cup outside of the *safe zone* [19] and therefore contributes to the potential risk of dislocation after THA [9]. In addition, the cup orientation was measured before and after the insertion of 2 screws to stabilize the cup. In some cases, the cup moved by a maximum of 2° after screws insertion. Also, visualizing the instruments in relation to the pelvis model on the screen in real-time motion provides a good way to asses the functional aspect of the joint. This may be relevant for surgeons who do not perform that specific type of surgery regularly.

## 5. Conclusion

When performing a total hip replacement, the use of a navigation device provides an additional tool in order to achieve a better implant positioning. In fact, postoperative CT-scans revealed that the final cup position during the surgery is in agreement with the orientation planned. Therefore, this validation study is relevant and indicates that the use of this navigation system is valid and precise to position the cup. Next, postoperative AP radiographs normally used in clinic are acceptable to determine the cup orientation, considering that knowing the patient's pelvis position on the table will decrease the error when determining the cup orientation from a 2D image, providing a crucial feedback to the surgical outcome.

## Figures and Tables

**Table 1:** Acetabular cup inclination (inc) and anteversion (ant) using postoperative CT-scan and AP radiograph images from 9 patients after THA.

Cup orientation values—Patient no.	Planned (pre-op) cup orientation (inc/ant)	Per-op cup orientation (Navitrack system readings) (inc/ant)	Postop CT-scan (inc/ant)	Δ between postop CT-scan and per-op reading (inc/ant)	Postop AP radiograph reading (inc/ant)	Δ between AP radiograph and postop CT-scan (inc/ant)
Patient 1	46/20	44/29	43/27	**−1/−2**	45/23	**2/−4**
Patient 2	46/20	45/22	47/21	**2/−1**	47/24	**0/3**
Patient 3	45/20	42/26	42/24	**0/−2**	46/28	**4/4**
Patient 4	50/25	52/24	50/22	**−2/−2**	52/24	**2/2**
Patient 5	49/25	50/23	47/20	**−3/−3**	50/23	**3/3**
Patient 6	45/21	43/25	41/23	**−2/−2**	43/23	**2/0**
Patient 7	48/22	46/30	43/30	**−3/0**	43/27	**0/−3**
Patient 8	51/23	60/25	56/25	**−4/0**	53/18	**−3/−7**
Patient 9	47/22	46/22	39/24	**−7/2**	37/20	**−2/−4 **

*Inclination Anteversion*	47 ± 2°	47 ± 6°	45 ± 5°	2.7 ± 2°	46 ± 5°	2.0 ± 1.3°
	22 ± 2°	25 ± 3°	24 ± 3°	1.6 ± 1°	23 ± 3°	3.3 ± 1.9°
(Average ± SD)						

## References

[1] Kennedy JG, Rogers WB, Soffe KE, Sullivan RJ, Griffen DG, Sheehan LJ (1998). Effect of acetabular component orientation on recurrent dislocation, pelvic osteolysis, polyethylene wear, and component migration. *Journal of Arthroplasty*.

[2] Schmalzried TP, Guttmann D, Grecula M, Amstutz HC (1994). The relationship between the design, position, and articular wear of acetabular components inserted without cement and the development of pelvic osteolysis. *Journal of Bone and Joint Surgery. Series A*.

[3] Wolf A, DiGioia AM, Mor AB, Jaramaz B (2005). Cup alignment error model for total hip arthroplasty. *Clinical Orthopaedics and Related Research*.

[4] Brien WW, Salvati EA, Wright TM, Burstein AH (1993). Dislocation following THA: comparison of two acetabular component designs. *Orthopedics*.

[5] Chandler DR, Glousman R, Hull D (1982). Prosthetic hip range of motion and impingement. The effects of head and neck geometry. *Clinical Orthopaedics and Related Research*.

[6] Cobb TK, Morrey BF, Ilstrup DM (1996). The elevated-rim acetabular liner in total hip arthroplasty: relationship to postoperative dislocation. *Journal of Bone and Joint Surgery. Series A*.

[7] Daly PJ, Morrey BF (1992). Operative correction of an unstable total hip arthroplasty. *Journal of Bone and Joint Surgery. Series A*.

[8] Honl M, Schwieger K, Salineros M, Jacobs J, Morlock M, Wimmer M (2006). Orientation of the acetabular component. A comparison of five navigation systems with conventional surgical technique. *Journal of Bone and Joint Surgery. Series B*.

[9] McCollum DE, Gray WJ (1990). Dislocation after total hip arthroplasty: causes and prevention. *Clinical Orthopaedics and Related Research*.

[10] Kelley TC, Swank ML (2009). Role of navigation in total hip arthroplasty. *Journal of Bone and Joint Surgery. Series A*.

[11] Ryan JA, Jamali AA, Bargar WL (2010). Accuracy of computer navigation for acetabular component placement in THA. *Clinical Orthopaedics and Related Research*.

[12] Blendea S, Eckman K, Jaramaz B, Levison TJ, Digioia AM (2005). Measurements of acetabular cup position and pelvic spatial orientation after total hip arthroplasty using computed tomography/radiography matching. *Computer Aided Surgery*.

[13] Amiot L-P, Lang K, Putzier M, Zippel H, Labelle H (2000). Comparative results between conventional and computer-assisted pedicle screw installation in the thoracic, lumbar, and sacral spine. *Spine*.

[14] Ackland MK, Bourne WB, Uhthoff HK (1986). Anteversion of the acetabular cup. Measurement of angle after total hip replacement. *Journal of Bone and Joint Surgery. Series B*.

[15] Murray DW (1993). The definition and measurement of acetabular orientation. *Journal of Bone and Joint Surgery. Series B*.

[16] DiGioia AM, Jaramaz B, Plakseychuk AY (2002). Comparison of a mechanical acetabular alignment guide with computer placement of the socket. *Journal of Arthroplasty*.

[17] Jaramaz B, Eckman K 2D/3D registration for measurement of implant alignment after total hip replacement.

[18] Babisch JW, Layher F, Amiot L-P (2008). The rationale for tilt-adjusted acetabular cup navigation. *Journal of Bone and Joint Surgery. Series A*.

[19] Lewinnek GE, Lewis JL, Tarr R (1978). Dislocations after total hip-replacement arthroplasties. *Journal of Bone and Joint Surgery. Series A*.

